# Prediction of robo-advisory acceptance in banking services using tree-based algorithms

**DOI:** 10.1371/journal.pone.0302359

**Published:** 2024-05-06

**Authors:** Witold Orzeszko, Dariusz Piotrowski

**Affiliations:** 1 Department of Applied Informatics and Mathematics in Economics, Nicolaus Copernicus University, Toruń, Poland; 2 Department of Financial Management, Nicolaus Copernicus University, Toruń, Poland; St John’s University, UNITED STATES

## Abstract

The banking sector is increasingly recognising the need to implement robo-advisory. The introduction of this service may lead to increased efficiency of banks, improved quality of customer service, and a strengthened image of banks as innovative institutions. Robo-advisory uses data relating to customers, their behaviors and preferences obtained by banks from various communication channels. In the research carried out in the work, an attempt was made to obtain an answer to the question whether the data collected by banks can also be used to determine the degree of consumer interest in this type of service. This is important because the identification of customers interested in the service will allow banks to direct a properly prepared message to a selected group of addressees, increasing the effectiveness of their promotional activities. The aim of the article is to construct and examine the effectiveness of predictive models of consumer acceptance of robo-advisory services provided by banks. Based on the authors’ survey on the use of artificial intelligence technology in the banking sector in Poland, in this article we construct tree-based models to predict customers’ attitudes towards using robo-advisory in banking services using, as predictors, their socio-demographic characteristics, behaviours and attitudes towards modern digital technologies, experience in using banking services, as well as trust towards banks. In our study, we use selected machine learning algorithms, including a decision tree and several tree-based ensemble models. We showed that constructed models allow to effectively predict consumer acceptance of robo-advisory services.

## 1. Introduction

The advances in digital technologies for data collection and processing observed in recent years have made robo-advisory one of the fastest growing services in the financial sector [1, 2]. Basing robo-advisory on artificial intelligence and machine learning eliminates or significantly reduces the need for human input [3]. Robo-advisory has been applied mainly in the relatively narrow area of making recommendations and investment decisions on financial instruments [4, 5]. This technology is used by people interested in automating the management of financial assets; they represent a relatively small proportion of financial institutions’ customers [6, 7]. Changing this state, according to experts, requires expanding the investment offering of robo-advisory [8]. The introduction of automation in the securities, banking and insurance sectors is also expected to increase the importance of this service [9–11].

A fundamental shift is now taking place in the use of robo-advisory, as this service is increasingly being introduced by banks [12]. On the one hand, they are faced with the challenge of a wide range of offered products as well as a huge number of customers with differing expectations and preferences. On the other hand, large-scale implementation of robo-advisory service can bring multi-dimensional and multifaceted benefits in the form of improved operational efficiency and increased profitability of banks’ business, support for financial institutions’ employees in giving advice, as well as wider access to financial services by customers [13–15]. The advantages of robo-advisory also include the reduction of conflicts of interest and of the negative impact of emotions and biases on financial decision-making [16].

Achieving the indicated benefits requires banks to take steps to attain a wider acceptance of robo-advisory among customers. This can best be achieved by addressing individuals potentially interested in robo-advisory. This, in turn, raises the need to chisel out target customers from among the general banking population. The literature identifies factors that significantly influence the acceptance of robo-advisory among consumers. Very often they refer to technological and operational aspects of robo-advisory: level of automation, transparency, and control [17], comprehensibility [18], perceived usefulness [19], privacy [20], perceived innovativeness [21], data security [22], ease of interaction and work efficiency [23–26]. Demographic and socio-economic factors are also important determinants of the acceptance of robo-advisory. The literature emphasizes the significance of consumer age and technological competences [27], as well as financial knowledge, literacy and experience [28–30]. Some studies demonstrate the importance of trust in the technologies used in robo-advisory and the providers of robo-advisory [4, 31–36], as well factors related to risk acceptance [37–39]. Limitations to the acceptance of robo-advisory services were also pointed out, related to reluctance to make decisions made by machines, especially when they contain an ethical component [40]. A study [41] also highlights the key importance of ethical issues for the acceptance of robo-advice, except that the variables used related to fairness in the area of the provision of financial services by banks. In the aforementioned study, the use of machine learning methods further showed that factors related to consumers’ belief in the benefits of implementing artificial intelligence technology in the banking sector were the most important determinants of robo-advice acceptance, while the least important variables were related to having a bank account, the consumer’s use of financial advice services and making financial investments on their own.

In addition to the mainstream research on the identification of robo-advisory acceptance factors, there are attempts in the literature to construct prediction models in this area [42]. In particular, models related to asset price forecasting and investment portfolio optimization were presented [43–47]. The proposed models are used to enhance forecasting accuracy and efficiency in robo-advisory. These models usually use market data related to the quotations of investment assets. Solutions taking into account macroeconomic variables [48], recommendations based on financial social networks [49], and social media sentiments [50] are also analyzed. To the best of the authors’ knowledge, only in one case did the prediction concern the acceptance of robo-advisory [51]. The linear regression models and four machine learning algorithms–namely, regression tree, random forest, gradient boosting and artificial neural network, were used in this work. The variables used in the prediction related to the demographic characteristics of respondents, investment experience, the degree of use of new information technologies, and, above all, expectations formulated towards robo-advisory services (prediction accuracy, transparency of the investment process, communication style with the investor, possibilities of interference in robo-advisor decisions). They showed that all machine learning algorithms showed superior prediction performance than linear regression.

The literature study on the acceptance of robo-advisory among consumers resulted in the identification of two research gaps. The first gap concerns the almost complete absence of prediction models related to the intention to use robo-advisory. The paper fills this gap by applying tree-based algorithms to predict robo-advisory acceptance in banking services. However, unlike the work [51], where the prediction of acceptance of robo-advisory services was based mainly on variables related to consumers’ expectations of said service, this study considers variables that banks are able to obtain in real conditions, in the customer service process. Collecting data on the use of financial services and the use of digital technologies in finance is relatively easy for banks and does not involve the need to conduct interviews with consumers, as is the case with data related to robo-advisory services. It was also found that robo-advisory has so far only been analysed in the context of financial instrument portfolio management. The work presented here fills this gap by extending robo-advisory research to other segments of the financial market. This is in line with the authors’ definition of robo-advisory, according to which robo-advisory is understood as automated advice on investing, saving, and obtaining finance using artificial intelligence technologies to make recommendations or relevant decisions, based on analysis of client and economic data.

The aim of the paper is to verify whether selected tree-based algorithms can be effectively applied to predict robo-advisory acceptance in banking services. The study carried out aims to verify the research hypothesis that the use of prediction models enables the correct identification of consumers interested in robo-advisory. The obtained results show that the predictive power of all applied models can be regarded as satisfying.

The next sections of this article are organised as follows. Section 2 presents the data obtained in the survey and the research methodology. Section 3 analyses the accuracy of the prediction models. Finally, Section 4 discusses the results and presents the main conclusions and practical implications.

## 2. Materials and methods

In our study we analyse the results of the survey on the use of artificial intelligence technology in the banking sector in Poland. This survey identifies the demographic and socio-economic characteristics, behaviours, and attitudes of consumers that determine respondents’ adoption of robo-advisory. The study was carried out based on the CATI method, using a questionnaire developed by the authors. In the first phase, a pilot survey was carried out to improve the efficiency of the main survey. The full-scale survey conducted in October 2020 covered a sample of 911 Polish citizens aged 18–65. The sample was representative of Polish society in terms of age, gender, and place of residence, the latter of which included the size of the town and the region of the country. A stratified random sampling technique was used to obtain a representative sample of the population. Based on Statistics Poland data, the structure of the population was characterised. Taking into account the above indicated characteristics, the population was divided into smaller subgroups known as strata. Subsequently, telephone numbers were randomly generated and called by the interviewer. Demographic data—age, gender, size of town, region of the country—assumed in the strata were then verified during the interview, followed by an invitation to a given person for a full interview, or termination of the interview, because the strata was already full.

The survey was conducted by professional research agency—Interactive Research Center—which was selected in a public procurement procedure. This agency is guided in its work by the International Code of ICC/ESOMAR, which ensures high quality of research and compliance with the principles of ethics. The participants of the study were people from the research panel who agreed to participate in research carried out periodically by the Interactive Research Center. Participation in our study was voluntary, and each respondent had the option to stop the study at any time. The study and the data obtained in the study were fully anonymous. Due to the fact that the research was non-interventional and did not have a clinical nature, the Research Ethics Committee of the Faculty of the Economic Science and Management at the Nicolaus Copernicus University in Toruń decided that ethical approval was not needed in this case.

Table 1 presents the variables used in the analysis and the structure of the responses given by the respondents. The dependent variable *Robo Intention* refers to respondents’ attitudes towards using robo-advisory that supports banking services in the following five years. The explanatory variables relate to the demographic and socio-economic characteristics of the respondents, in particular their experience in using banking services, their attitudes towards modern digital technologies (including perceptions of artificial intelligence and robo-advisory in banking), and trust towards banks.

**Table 1:** Characteristics of variables and the structure of responses obtained by the computer-assisted telephone interview survey (*N* = 911).

Variable	Variable description	Responses	%
Robo Intention	Intention of use robo-advisory in banks’ services in next 5 years -response variable	Definitely not	18.8
		Rather not	28.1
		It’s hard to say	29.1
		Rather yes	20.0
		Definitely yes	4.0
Gender	Gender	Female	50.2
		Male	49.8
Age Group	Age group	18–24	8.0
		25–34	24.1
		35–44	25.3
		45–54	19.6
		55–65	23.0
Residence	Place of residence	Village	28.8
		Village-suburban area	7.9
		City up to 20,000	13.3
		City up to 100,000	20.2
		City up to 500,000	17.8
		City over 500,000	12.0
Education	Education level	Primary and below	2.0
		Lower secondary	18.5
		and basic vocational	
		Secondary	40.4
		Higher	39.1
Bank Account	Bank account possession	No	5.4
		Yes	94.6
Investment Advisory	Use of bank advisory services related to savings and investment	No	66.5
		Yes	33.5
Loan Advisory	Use of bank advisory services related to obtaining financing in the form of a loan	No	46.1
		Yes	53.9
Financial Advisory	Use of bank advisory services related to savings, investment, or obtaining financing in the form of a loan	No	35.2
		Yes	64.8
Own Investments	Experience of investing independently in the financial market	No	78.2
		Yes	21.8
Internet Use	Frequency of using Internet	No or less than once a year	3.7
		Several times a year	1.4
		Several times a month	6.4
		A few times a week	12.7
		Several times a day	75.8
Social Media Use	Frequency of using social media	No or less than once a year	22.5
		Several times a year	1.3
		Several times a month	5.9
		A few times a week	15.2
		Several times a day	55.1
Internet Banking Use	Frequency of using Internet banking services	No or less than once a year	18.9
		Several times a year	2.3
		Several times a month	23.3
		A few times a week	40.4
		Several times a day	15.1
Mobile Banking Use	Frequency of using mobile banking services	No or less than once a year	44.0
		Several times a year	0.8
		Several times a month	9.6
		A few times a week	26.5
		Several times a day	19.1
E-banking Use	Frequency of using e-banking services (Internet or mobile banking)	No or less than once a year	15.5
		Several times a year	0.9
		Several times a month	18.7
		A few times a week	40.0
		Several times a day	24.9
Bank AI Experience	Experience in using AI in banks’ services (chatbots and robo-advisors)	No	77.9
		Yes	22.1
Non-banking AI Experience	Experience in using AI in non-banking services	No	49.6
		Yes	50.4
Test New Technology	Passion for testing new technological solutions, devices and applications	Definitely not	6.7
		Rather not	18.7
		It’s hard to say	17.8
		Rather yes	39.6
		Definitely yes	17.2
AI Preferences	Agree with statement: The use of artificial intelligence by banks to analyze personal and financial data may allow better understanding of customer expectations and preferences	Definitely not	8.8
		Rather not	12.4
		It’s hard to say	21.8
		Rather yes	38.9
		Definitely yes	18.1
AI Quality	Agree with statement: The use of artificial intelligence technologies by banks will increase the quality of services provided	Definitely not	7.0
		Rather not	15.0
		It’s hard to say	31.8
		Rather yes	34.2
		Definitely yes	12.0
Trust	Agree with statement: Banks are trustworthy institutions	Definitely not	2.5
		Rather not	9.2
		It’s hard to say	18.5
		Rather yes	58.4
		Definitely yes	11.4
Ethics	Agree with statement: Banks operating in Poland comply with ethical standards in relations with their customers	Definitely not	3.4
		Rather not	11.3
		It’s hard to say	18.8
		Rather yes	56.0
		Definitely yes	10.5
Honest Advisory	Agree with statement: Banks advise honestly	Definitely not	3.5
		Rather not	12.9
		It’s hard to say	26.0
		Rather yes	50.0
		Definitely yes	7.6
Unwanted Products	Agree with statement: Banks offer products that do not meet customers’ needs	Definitely not	5.2
		Rather not	16.5
		It’s hard to say	19.1
		Rather yes	38.9
		Definitely yes	20.3
Manipulate Information	Agree with statement: Banks manipulate information about financial services	Definitely not	6.2
		Rather not	27.6
		It’s hard to say	29.9
		Rather yes	26.6
		Definitely yes	9.7
Lack Complete Information	Agree with statement: Banks operating in Poland do not provide full information on financial products	Definitely not	6.6
		Rather not	33.0
		It’s hard to say	26.8
		Rather yes	26.0
		Definitely yes	7.6
Personal Data Use	Agree with statement: Banks handle the personal data of clients properly	Definitely not	3.8
		Rather not	9.9
		It’s hard to say	19.5
		Rather yes	51.6
		Definitely yes	15.1
Data Ethics	Agree with statement: It is ethical for banks to obtain information about customers from photos, videos, blogs and forums made available by them publicly on social media	Definitely not	42.7
		Rather not	29.8
		It’s hard to say	17.1
		Rather yes	8.2
		Definitely yes	2.2
Data Sharing	Willingness to consent to the bank’s analysis of the content posted on a public profile in social media	Definitely not	71.7
		Rather not	16.4
		It’s hard to say	7.6
		Rather yes	3.0
		Definitely yes	1.3

Source: Own research.

In our study we build predictive models for the response variable *Robo Intention* using all the other variables from Table 1 as predictors. Since this variable is categorical (it takes five ordinal values–see Table 1), we created classification models, where each of five predicted classes refer to a group of responders with a specific attitude to use robo-advisory.

Tree-based methods are popular machine-learning methods for data prediction and exploratory which have some good advantages over traditional statistical methods. They stratify the predictor space into a number of simple regions and then fit a simple model (like a constant one) in each of them [52–54]. The process of constructing a classification tree involves two main steps [55]:

1. Dividing the predictor space (i.e. the set of possible values for X_1_,X_2_,…,X_p_) into M distinct and non-overlapping regions R_1_,R_2_,…,R_M_ (corresponding to the nodes of the tree).
2. Calculating the predicted class as the majority class in this region.

In our research, we build classification trees using the CART algorithm [56] in Matlab R2020. Optimal regions R_1_,R_2_,…,R_M_ are determined in the following steps [57]:

1. Start with all input data, and examine all possible binary splits on every predictor.
2. Select a split with the least value of the Gini’s diversity index.
3. Impose the split.
4. Repeat recursively for the two child nodes.

Splitting continues until one of the following stopping rule is triggered:

- The node contains only observations of one class.
- There are fewer than *MinParentSize* = 10 observations in this node.
- Any split imposed on this node produces children with fewer than *MinLeafSize* = 1 observations.
- The algorithm splits *MaxNumSplits* = *N*-1 nodes (where *N* is the training sample size).

Apart from a single classification tree we additionally apply several tree-based ensemble models. The idea of ensemble modeling is to aggregate two or more models to obtain predictions. This approach often provides higher accuracy and lower prediction variance compared to a single tree model while maintaining some of the beneficial qualities of tree models (e.g. ability to interpret relationships between predictors and outcome) [58–60].

In order to build tree-based ensemble models we apply bagging and boosting techniques. Both of them improve the predictive performance of tree models, however bagging turns out to be a variance reduction scheme and boosting primarily reduce the model bias [61].

Bagging (*bootstrap aggregation*) introduced by Breiman [62] is a smoothing technique which consists in bootstrapping in conjunction with any regression or classification model to construct an ensemble model. According to this approach, an ensemble of decision trees is built for bootstrap samples, i.e. for samples created by a random selection (without replacement) of the instances from the training set. The predictions from the trees are averaged in order to give the bagged model’s prediction. Bühlmann and Yu [63] showed that the bagging technique reduces a variance and the mean squared error. In our study we applied the random forest algorithm which combines bagging with feature randomization [58]. According to this approach, in addition to using different subsets of the data, random forest introduces randomness in the selection process of explanatory variables for each tree [64]. At each node of a decision tree, a random subset of variables is considered for splitting, which helps in creating diverse and less correlated trees.Apart from bagging, we also apply three algorithms of boosting for multiclass classification: adaptive boosting (AdaBoost.M2 [65]), random undersampling boosting (RUS-Boost [66]) and totally corrective boosting (TotalBoost [67]). The idea of the boosting techniques is to repeatedly run a weak learner on various distributed training data. The classifiers produced by the weak learners are then combined into a single composite strong classifier in order to achieve a higher accuracy than the individual trees would be capable of [68]. In effect, in contrast to bagging which is a parallel ensemble algorithm, boosting belongs to sequential ensemble methods. Moreover, it is worth mentioning that the boosting procedure can be viewed as a nonparametric optimization algorithm in function space and has been empirically demonstrated to be very accurate in case of classification tasks [61].

In our study, the whole dataset (911 responses) was randomly divided into the training and testing sets of, respectively, 731 and 180 responses. The training set was used to build the models and to optimize them by tuning their parameters. In order to tune the hyperparameters, we applied 5-fold cross-validation procedure with the Bayesian optimization (e.g. [69]) to minimize the cross-validation loss. The test set was used to assess the prediction accuracy. For this purpose we applied two approaches. First, we calculated the Kendall’s tau and Spearman’s rank correlation coefficients between predicted and actual values of the response variable. Next, we calculated confusion matrices, which represent counts from predicted and actual values of this variable.

## 3. Results

Table 2 presents the Kendall’s tau and Spearman’s rank correlation coefficients between predicted and actual values of the response variable *Robo Intention*. It also contains the *p*-values for testing the hypothesis of no correlation.

**Table 2:** Correlation coefficients between predicted and actual values.

	CART	Random forest	AdaBoost	RUS-Boost	TotalBoost
Kendall’s tau /p-value/	0.2473 /0.0001/	0.3129 /0.0000/	0.3990 /0.0000/	0.2322 /0.0001/	0.2478 /0.0001/
Spearman’s correlation /p-value/	0.2925 /0.0001/	0.3602 /0.0000/	0.4616 /0.0000/	0.2795 /0.0001/	0.2884 /0.0001/

It is clearly seen that all coefficients are moderate, however they are strongly significant. The most accurate predictions were obtained from the adaptive boosting algorithm. Moreover, we can see that random forest leads to better results than three other algorithms: CART, random undersampling boosting and totally corrective boosting.

Next we constructed confusion matrices (see Tables 3–7), where each row represents the instances in an actual class, while columns represent the instances in a predicted class. Each cell in the confusion matrix contains the number of instances and their percentage in relation to the total number of all instances in the actual class. It means that the predicted value is accurate when it aligns with the corresponding actual value, and the efficacy of the predictive model is accentuated when a higher percentage of instances align along the diagonal cells. To enhance visibility, these cells have been highlighted with a dark background. However, it is crucial to acknowledge that the target variable is ordinal, which implies that in case of incorrect prediction, the proximity of the predicted class to the actual one becomes significant. To account for this, cells representing predictions that, while not perfect, indicate the nearest adjacent class have been shaded with a light gray color. Consequently, instances within these cells epitomize predictions that can be also deemed as satisfactory, considering their proximity to the actual class.

**Table 3:** Confusion matrix for CART.

Actual	1	14 (37.8%)	13 (35.1%)	8 (21.6%)	1 (2.7%)	1 (2.7%)
	2	7 (15.6%)	18 (40.0%)	14 (31.1%)	6 (13.3%)	0 (0.0%)
	3	9 (19.6%)	12 (26.1%)	18 (39.1%)	7 (15.2%)	0 (0.0%)
	4	9 (21.4%)	7 (16.7%)	14 (33.3%)	11 (26.2%)	1 (2.4%)
	5	0 (0.0%)	2 (20.0%)	3 (30.0%)	5 (50.0%)	0 (0.0%)
		1	2	3	4	5
	Predicted					

**Table 4:** Confusion matrix for random forest.

Actual	1	4 (10.8%)	19 (51.4%)	11 (29.7%)	3 (8.1%)	0 (0.0%)
	2	1 (2.2%)	19 (42.2%)	18 (40.0%)	7 (15.6%)	0 (0.0%)
	3	0 (0.0%)	15 (32.6%)	26 (56.5%)	5 (10.9%)	0 (0.0%)
	4	1 (2.4%)	8 (19.0%)	17 (40.5%)	16 (38.1%)	0 (0.0%)
	5	0 (0.0%)	1 (10.0%)	5 (50.0%)	4 (40.0%)	0 (0.0%)
		1	2	3	4	5
	Predicted					

**Table 5:** Confusion matrix for AdaBoost.

Actual	1	13 (35.1%)	12 (32.4%)	9 (24.3%)	3 (8.1%)	0 (0.0%)
	2	2 (4.4%)	14 (31.1%)	19 (42.2%)	10 (22.2%)	0 (0.0%)
	3	0 (0.0%)	10 (21.7%)	24 (52.2%)	12 (26.1%)	0 (0.0%)
	4	1 (2.4%)	5 (11.9%)	17 (40.5%)	19 (45.2%)	0 (0.0%)
	5	0 (0.0%)	0 (0.0%)	4 (40.0%)	6 (60.0%)	0 (0.0%)
		1	2	3	4	5
	Predicted					

**Table 6:** Confusion matrix for RUS-Boost.

Actual	1	13 (35.1%)	12 (32.4%)	6 (16.2%)	4 (10.8%)	2 (5.4%)
	2	10 (22.2%)	10 (22.2%)	14 (31.1%)	6 (13.3%)	5 (11.1%)
	3	5 (10.9%)	10 (21.7%)	16 (34.8%)	12 (26.1%)	3 (6.5%)
	4	3 (7.1%)	12 (28.6%)	9 (21.4%)	16 (38.1%)	2 (4.8%)
	5	1 (10.0%)	3 (30.0%)	0 (0.0%)	2 (20.0%)	4 (40.0%)
		1	2	3	4	5
	Predicted					

**Table 7:** Confusion matrix for TotalBoost.

Actual	1	6 (16.2%)	9 (24.3%)	14 (37.8%)	8 (21.6%)	0 (0.0%)
	2	3 (6.7%)	12 (26.7%)	19 (42.2%)	11 (24.4%)	0 (0.0%)
	3	1 (2.2%)	9 (19.6%)	22 (47.8%)	14 (30.4%)	0 (0.0%)
	4	1 (2.4%)	5 (11.9%)	19 (45.2%)	17 (40.5%)	0 (0.0%)
	5	0 (0.0%)	0 (0.0%)	3 (30.0%)	7 (70.0%)	0 (0.0%)
		1	2	3	4	5
	Predicted					

Based on the confusion matrices, we calculated the overall accuracy of the models (see Table 8), i.e. the fraction of the responders from the testing sample that were correctly classified. Additionally, we extended our calculations by counting samples which were classified not only to the actual but also to the adjacent class (i.e. the instances which are located in the cells marked by both–the dark and light grey background). These extended accuracies are presented in Table 8 in brackets.

**Table 8:** Accuracy of predictions.

	CART	Random forest	AdaBoost	RUS-Boost	TotalBoost
Accuracy	33.9% (77.2%)	36.1% (82.8%)	38.9% (85.0%)	32.8% (75.0%)	31.7% (78.9%)

The results from the confusion matrices confirm the conclusions from the correlation coefficients–the most accurate predictions are obtained from the adaptive boosting algorithm and random forest has an advantage over three other algorithms: CART, random undersampling boosting and totally corrective boosting. However, generally, the obtained results show that accuracy of all the applied models is not very high. Their overall accuracy does not exceed 40%, however it is significantly higher than accuracy of a random model (with expected accuracy at 20% level). Moreover, accuracy of the models increases if we take into account also predictions which are close to the actual values, i.e. they indicate the adjacent class. In this case, the overall accuracy is about 80%.

## 4. Discussion and concluding remarks

The literature studies have shown that the use of prediction models determining consumers’ intentions to use robo-advisory services has so far attracted insufficient interest from the world of science. Achieving the aim of the work, which was to construct tree-based models and to test their effectiveness in predicting the acceptance of robo-advisors in banking services, made it possible to fill the diagnosed research gap. In our work, we applied selected algorithms, including a decision tree and several ensemble tree-based algorithms–random forest, adaptive boosting, random undersampling boosting, and totally corrective boosting. The obtained results show that all of the applied models can be regarded as useful, because they allow the prediction of consumer acceptance of robo-advisory services with satisfactory accuracy. The most accurate predictions were obtained from the adaptive boosting algorithm and random forest had an advantage over three other algorithms: CART, random undersampling boosting, and totally corrective boosting.

The expected development of robo-advisory services in various segments of the financial market indicates the need to take actions aimed not only at the development of digital technologies, especially artificial intelligence, but also at identifying the needs and expectations of consumers. The predictive models proposed in the paper can be used by banks to identify customers interested in robo-advisory. Identifying potential users of robo-advisory services is helpful in further marketing activities. Directing the message to a pre-defined group of consumers may bring benefits desired from the bank’s point of view in the form of cost reduction and increased efficiency of undertaken activities. It can also be interpreted as attention to meeting consumer needs and a manifestation of innovativeness of financial institutions.

The second important practical application of the results relates to the type of customer data processed. In our study, we applied the data obtained from the authors’ survey on the use of artificial intelligence technology in the banking sector in Poland. In order to predict customers’ attitudes towards using robo-advisory in banking services, we used 28 various predictors referring to their demographic and socio-economic characteristics, behaviours, attitudes towards modern digital technologies, and experience in using banking services. It should be noted, that predictors are universal and can be used in relation to clients from other countries with a developed banking sector in which remote channels of communication with clients are widely used. Another advantage of our study is the relative ease of obtaining the data used. Banks have access to customers’ personal data, are able to determine the type and frequency of use of banking products, preferences in terms of communication channels with the bank, and also know consumers’ payment method habits. The study consciously refrained from using variables expressing consumers’ attitudes towards specific features of robo-advisory services, because obtaining this type of data by banks does not occur naturally during customer service, but requires an interview dedicated to this purpose. Gaining knowledge about consumers’ expectations towards robo-advice in this way means that the validity of creating prediction models regarding the intention to adopt robo-advice based on the collected data is limited.

The widespread use of prediction models related to customer behavior by banks may improve the quality of financial services. However, these activities require the collection of the right type and amount of data. The use of digital technologies in customer service significantly facilitates and improves the efficiency of data collection, analysis and conclusions based on them. The work presented here confirms the practical applicability of prediction, but also highlights the relevance of the issue of processing data on market phenomena and consumers behavior to modern finance.

The results obtained in the study affirm the potential for effectively forecasting consumer acceptance of robo-advisory services using tree-based methods. Future research endeavors in this domain could notably expand by incorporating alternative predictive techniques, encompassing various machine learning algorithms. Additionally, exploration should extend to the inclusion of alternative predictors representing other respondent characteristics that might influence their attitudes toward robo-advisory services. An important avenue for future investigations also appears to be the determination of the universality of predictive models for the acceptance of robo-advisory services. This may involve assessing the extent to which their effectiveness is contingent on specific contextual factors, such as the regulatory environment or cultural considerations in particular countries. It is crucial to underscore that with the dynamic evolution of financial services and the consequent rapid changes in the awareness and attitudes of bank clients, such models may face swift obsolescence. This underscores the necessity for continual monitoring of their relevance and actuality by financial institutions utilizing them.
